# Developing the Lung Graph-Based Machine Learning Model for Identification of Fibrotic Interstitial Lung Diseases

**DOI:** 10.1007/s10278-023-00909-7

**Published:** 2024-01-16

**Authors:** Haishuang Sun, Min Liu, Anqi Liu, Mei Deng, Xiaoyan Yang, Han Kang, Ling Zhao, Yanhong Ren, Bingbing Xie, Rongguo Zhang, Huaping Dai

**Affiliations:** 1 National Center for Respiratory Medicine, State Key Laboratory of Respiratory Health and Multimorbidity; National Clinical Research Center for Respiratory Diseases；Institute of Respiratory Medicine, Chinese Academy of Medical Sciences; Department of Pulmonary and Critical Care Medicine, China-Japan Friendship Hospital, Beijing, 100029 China; 2 Department of Medical Oncology, State Key Laboratory of Oncology in South China, Guangdong Key Laboratory of Nasopharyngeal Carcinoma Diagnosis and Therapy, Collaborative Innovation Center for Cancer Medicine, Sun Yat-Sen University Cancer Center, Guangzhou, Guangdong Province, 510060 China; 3 Department of Radiology, China-Japan Friendship Hospital, Beijing, 100029 China; 4 Chinese Academy of Medical Sciences and Peking Union Medical College, Beijing, 100730 China; 5 Institute of Advanced Research, Infervision Medical Technology Co., Ltd., Beijing, 100025 China; 6 Department of Clinical Pathology, China-Japan Friendship Hospital, Beijing, 100029 China; 7 Capital Normal University, Beijing, 100048 China

**Keywords:** Fibrotic interstitial lung disease, Machine learning, Lung graph, High-resolution computed tomography

## Abstract

Accurate detection of fibrotic interstitial lung disease (f-ILD) is conducive to early intervention. Our aim was to develop a lung graph-based machine learning model to identify f-ILD. A total of 417 HRCTs from 279 patients with confirmed ILD (156 f-ILD and 123 non-f-ILD) were included in this study. A lung graph-based machine learning model based on HRCT was developed for aiding clinician to diagnose f-ILD. In this approach, local radiomics features were extracted from an automatically generated geometric atlas of the lung and used to build a series of specific lung graph models. Encoding these lung graphs, a lung descriptor was gained and became as a characterization of global radiomics feature distribution to diagnose f-ILD. The Weighted Ensemble model showed the best predictive performance in cross-validation. The classification accuracy of the model was significantly higher than that of the three radiologists at both the CT sequence level and the patient level. At the patient level, the diagnostic accuracy of the model versus radiologists A, B, and C was 0.986 (95% CI 0.959 to 1.000), 0.918 (95% CI 0.849 to 0.973), 0.822 (95% CI 0.726 to 0.904), and 0.904 (95% CI 0.836 to 0.973), respectively. There was a statistically significant difference in AUC values between the model and 3 physicians (*p* < 0.05). The lung graph-based machine learning model could identify f-ILD, and the diagnostic performance exceeded radiologists which could aid clinicians to assess ILD objectively.

**Graphical Abstract:**

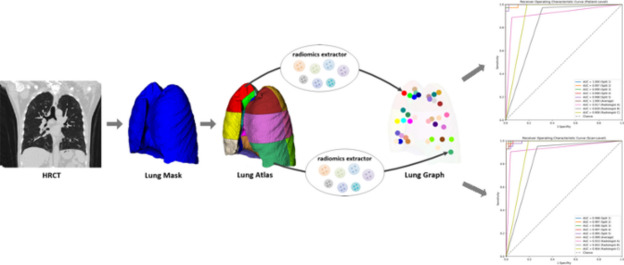

Given a sequence of HRCT slices from a patient, the lung field is first automatically extracted. Next, this lung region is divided into 36 sub-regions using geometric rules, obtaining a lung atlas. And then, the lung graph is built based on 3D radiomics features of each sub-region of the lung atlas. Finally, the model’s predictions were compared to the physicians’ assessment results.

## Introduction

Interstitial lung diseases (ILDs) are a group of heterogeneous diseases caused by various causes of alveolar inflammation and/or fibrosis [1, 2]. The degree and distribution of inflammation and fibrosis vary among different types of ILDs and at different development stages. Fibrotic interstitial lung diseases (f-ILDs) are the end-stage of ILD [3]. Idiopathic pulmonary fibrosis (IPF) is the classic form of f-ILD with a sustained progressive phenotype which manifests as fibrosis at an early stage [4–9]. As the most common form of f-ILD, the imaging and pathological histology of IPF present as usual interstitial pneumonia (UIP) [2, 10–12]. Whereas hypersensitivity pneumonitis (HP), nonspecific interstitial pneumonia (NSIP), and connective tissue disease–associated interstitial lung disease (CTD-ILD) present predominantly with inflammation in the early stages; fibrosis develops and gradually worsens with disease progression to f-ILD [13–16]. Fibrotic ILD is often accompanied by progressive lung structural destruction and decreased lung function [17, 18].

Despite the diversity of f-ILD classes and the difficulty of clinical management, these patients have similarities in clinical course, imaging, treatment, and prognosis, and the disease continues to progress [19–22]. Early and rapid identification of f-ILD is particularly important to improve the long-term survival and prognosis. HRCT is a key method to demonstrate the characteristics of ILD including non-f-ILD and f-ILD [19], which mainly present as a reticular pattern, traction bronchiectasis with or without honeycombing on HRCT. However, its evaluation extremely depends on the experience of radiologist and is time-consuming for the large amount of data on HRCT. The graph model is a complete framework that was first proposed for brain connectivity analysis, which divides the brain into a fixed number of anatomical regions and compares the neural activity in different regions. The method was subsequently improved by Dicente Cid et al. who proposed a more complex graph model for lung diseases. This 3D lung texture–based structural analysis has achieved better results in the classification of tuberculosis, early identification of multi-drug-resistant tuberculosis, and identification of pulmonary hypertension and pulmonary embolism [23, 24]. Thus, our objective is to apply this 3D lung texture–based structural analysis to assist in identification of f-ILD.

## Materials and Methods

### Study Cohort and Design

**Fig. 1:**
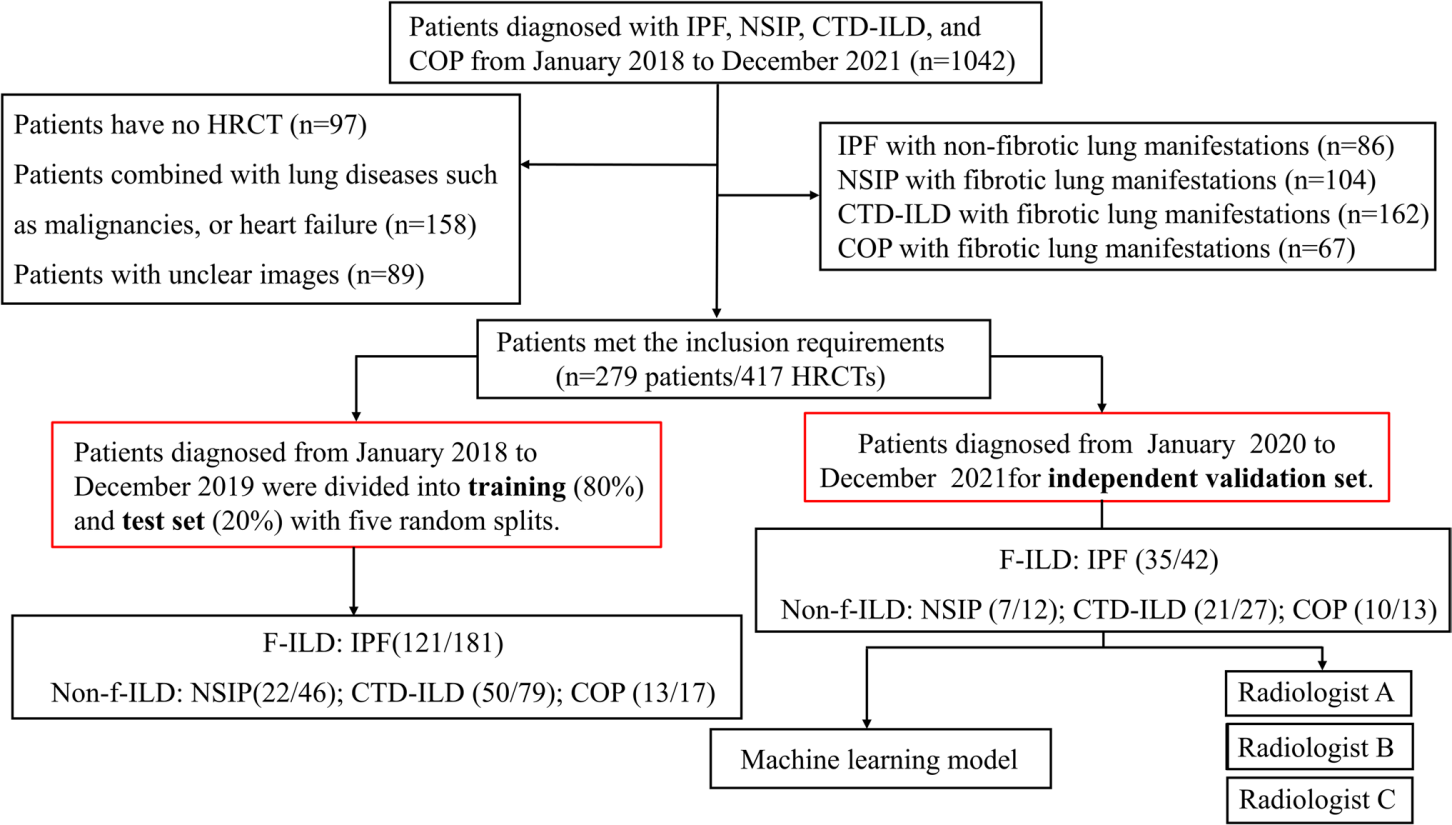
Flow chart and study design. IPF, idiopathic pulmonary fibrosis; NSIP, nonspecific interstitial pneumonia; CTD-ILD, connective tissue disease-associated interstitial lung disease; COP, cryptogenic organizing pneumonia

This lung graph–based machine learning study based on an ILD cohort was performed according to the Declaration of Helsinki and was approved by the ethics committee of our hospital (NO. 2017–25). Written consent of individuals was obtained. We retrospectively extracted 417 HRCTs of 279 patients with ILD at our hospital from January 2018 to December 2021. The determination of ILD was made by a multidisciplinary team (MDT) including at least one attending physician in Pulmonary and Critical Care Medicine, especially a specialist in ILD, two specialists with more than 10 years of experience in chest radiology, and one lung pathologist with 10 years of experience in lung pathology according to the diagnostic guidelines [1, 6]. All patients suspected of ILD experienced standard diagnostic procedures for ILD in our center, including detailed investigation of medical history, clinical symptoms and physical examination, laboratory tests for routine, connective tissue diseases, pulmonary function tests (PFTs), HRCT, bronchoalveolar lavage, and/or transbronchial lung biopsy or transbronchial lung cryobiopsy, sometimes video-assisted thoracoscopic surgery or surgical lung biopsy depending on the clinical requirements and multidisciplinary discussion [25]. The patients were treated by guidelines or consensus recommended and followed up by a clinic visit per 3 to 6 months or at time on clinical requirement. Each patient completed 1 to 5 HRCT scans. Cases diagnosed from January 2018 to December 2019 were used to train and test the model. Cases from January 2020 to December 2021 were used for independent validation and also visual assessment by radiologists. The detailed screening flow chart is shown in Fig. 1. The inclusion criteria are as follows: (1) patients with ILD diagnosed during the study period, (2) they received at least one supine HRCT sequence. The exclusion criteria are as follows: (1) significant respiratory motion artifacts on HRCT, (2) comorbidity with malignancy or other lung diseases, such as emphysema, (3) combined heart failure.

Patients with radiological and/or pathological evidence of fibrosis and a restrictive physiologic impairment pattern of FVC < 80% prediction were defined as f-ILD. The fibrotic patterns on HRCT were defined as reticulations, interlobular septal thickening, lung architectural distortion, honeycombing, and traction bronchiectasis. The fibrotic histologic findings included alveolar and interlobular septal thickening, fibroblast proliferation with collagen deposition, and architectural distortion. The flow chart of ILD diagnosis and study design is shown in Fig. 1.

### Pulmonary Function Tests

All patients underwent PFTs (MasterScreen, Vyaire Medical GmbH, Hochberg, Germany). PFT measurements included the percentage of predicted forced vital capacity (FVC%), percentage of forced expiratory volume in one second (FEV1%), FEV1/FVC%, percentage of predicted total lung capacity (TLC%), and percentage of predicted DLco corrected for the measured hemoglobin (DLco%).

### CT Protocol

All patients were scanned in the supine position on a multilayer spiral CT device (Lightspeed VCT/64, GE Healthcare; Toshiba Aquilion ONE TSX-301C/320; Philips iCT/256; Siemens FLASH Dual Source CT) at the end of inspiration scanning from the lung apex to the lung base. Acquisition and reconstruction parameters for HRCT sequences included tube voltage of 100–120 kV, tube current of 100–300 mAs, slice thickness of 0.625–1 mm, reconstruction increments of 1–1.25 mm, table speed of 39.37 mm/s, and gantry rotation time of 0.8 s.

### Lung Atlas Segmentation

All CT scans were first resampled into isometric voxels with a voxel size of 1 mm in all three dimensions. After this pre-processed step, a pipeline consisting of two steps was performed to obtain a specific human lung per CT scan, regarded as the lung atlas. As the first step, the lung fields were automatically extracted by a deep learning–based segmentation method (i.e., U-Net) provided by a medical imaging solution software (InferRead™ CT Lung, version R3.12.3; Infervision Medical Technology Co., Ltd., Beijing, China). And then, each lung mask was divided into 36 sub-regions via several geometric rules, obtaining a lung atlas.

### Radiomics-based Lung Graph Model Construction

Given a division of the lung with *N* regions *r* = {*r*_*1*_, *r*_*2*_, …, *r*_*N*_}, for a radiomics feature *f*, we define a graph model *G*_*F*_ of the lung based on that feature as the set of *N* regional feature nodes *F* = {*f*_*1*_, *f*_*2*_, …, *f*_*N*_}.

For each sub-region *r*_*i*_ from a given lung atlas, 1004 radiomics features were extracted: 187 first-order statistical features, 14 three-dimensional shape features, 253 GLCM features, 176 GLRLM features, 165 GLSZM features, 55 NGTDM features, and 154 GLDM features. This feature extraction procedure was done by using an open-source PyRadiomics software package (version 3.0.1; https://pyradiomics.readthedocs.io) in the Python environment (version 3.7.3; https://www.python.org/).

**Fig. 2:**
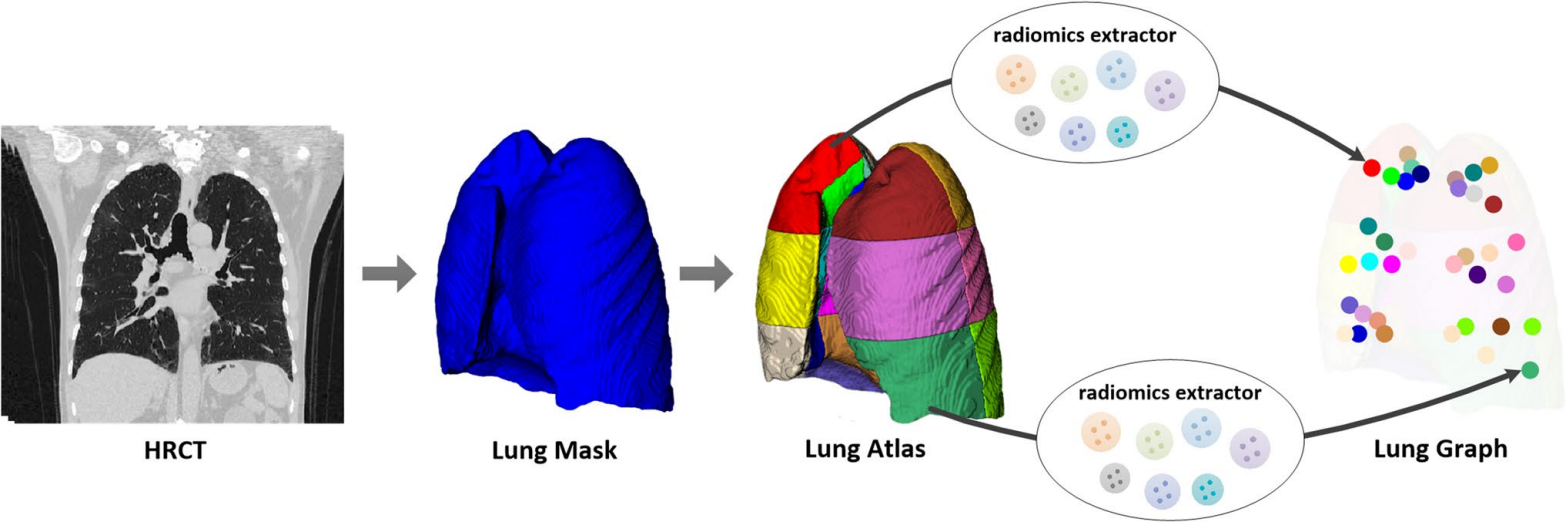
The construction flow of the lung graph. Given a sequence of HRCT slices from a patient, the lung field is first automatically extracted. And then, this lung region is divided into 36 sub-regions using geometric rules, obtaining a lung atlas. Finally, the lung graph is built based on 3D radiomics features of each sub-region of the lung atlas

**Fig. 3:**
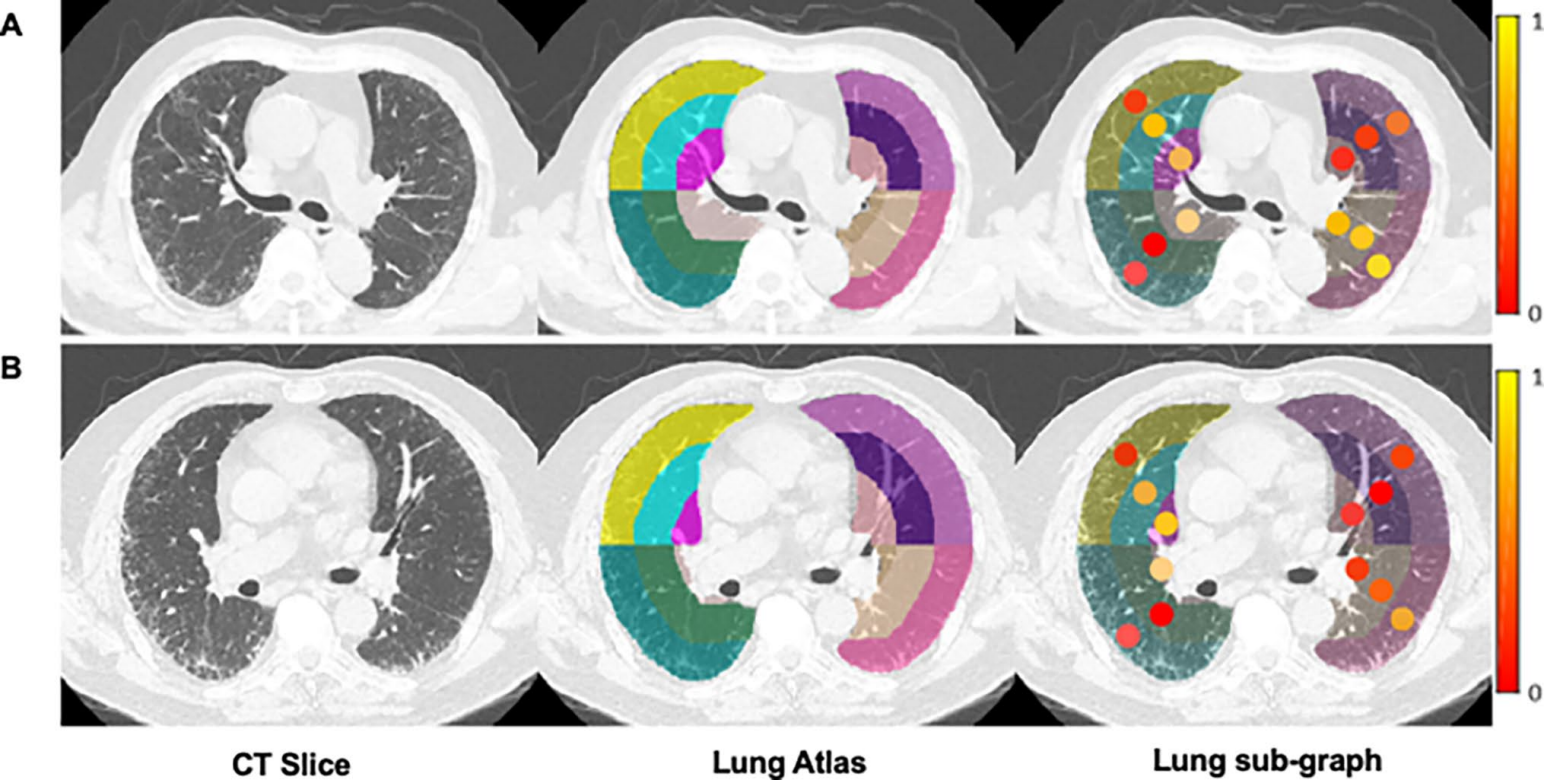
Two examples of lung graph construction. **A** A patient (male, age 78 years old) with non-fibrotic interstitial lung disease (NSIP). **B** A patient (male, age 70 years old) with fibrotic interstitial lung disease (idiopathic pulmonary fibrosis). From left to right: cropped CT slices of these two patients, the automatically generated lung atlas slices, the corresponding lung subgraph. This subgraph was obtained by the radiomics feature called log_sigma-1–0-mm-3D_glszm_SizeZoneNonUniformity. Each dots represents the strength of that feature in the subgraph. For better visualization, the values of each sub-region were scaled to (0,1)

Using the obtained lung atlas and the extracted features, 1004 radiomics-based lung graph models were established, and each graph model contained 36 feature nodes. (Figs. 2 and 3: The construction flow of the lung graph).

### Graph-based Lung Descriptor Generation

Considering data derived from patients who have undergone lung resection or with lung shrinking, the number of the nodes in the lung graph was 36 at most. Hence, a fixed number of statistics were selected to describe the distribution of each graph in order to compare the differences between different patients.

Ten statistics were calculated for each lung graph *G*_*F*_ in this study, including maximum value (s_1_(*G*_*F*_) = max(*G*_*F*_)), minimum value (s_2_(*G*_*F*_) = min(*G*_*F*_)), median value (s_3_(*G*_*F*_) = median(*G*_*F*_)), the 10th percentile value (s_4_(*G*_*F*_) = percentile(*G*_*F*_,10)), the 90th percentile value (s_5_(*G*_*F*_) = percentile(*G*_*F*_,90)), mean value (s_6_(*G*_*F*_) = mean(*G*_*F*_)), standard deviation (s_7_(*G*_*F*_) = std(*G*_*F*_)), interquartile range (s_8_(*G*_*F*_) = percentile(*G*_*F*_,75)-percentile(*G*_*F*_,15)), skewness (s_9_(*G*_*F*_) = skew(*G*_*F*_)), and kurtosis (s_10_(*G*_*F*_) = kurt(*G*_*F*_)). And then, the graph-based lung descriptor was defined as the following vector:

*s*(*G*_*F*_) = (*s*_1_(*G*_*F*_), *s*_2_(*G*_*F*_),…, *s*_10_(*G*_*F*_)) ∈ ℝ.^10^

As mentioned above, 1004 radiomics-based lung graphs were generated, so the final lung descriptor in our study was defined as the concatenation of the 1004 graph-based lung descriptors:

*S* = (*s*(*G*_*F1*_) || *s*(*G*_*F2*_) || … || *s*(*G*_*F1004*_)) ∈ *ℝ*.^10040^

### Dimensionality Reduction on Lung Descriptor

The dimension of the lung descriptor is massive, leading easily to the overfitting problem. To avoid this problem, a two-step flow for dimensionality reduction was adopted before the training model. Firstly, the Mann–Whitney *U* test was done between fibrotic and non-fibrotic ILD patients for all elements in the graph-based lung descriptor. Ranking these elements according to the obtained *p*-values in ascending order, the top 1% of the sorted ones were retained and fed into the subsequent step. Secondly, the Pearson correlation coefficient (*r*) was calculated between each pair of the inputted features. If the absolute *r* of a pair of features was greater than 0.85, the feature with the larger *p*-value from the abovementioned test in this pair was removed from the feature set.

### Machine Learning Model Development and Validation

Using the dimensionality-reduced lung descriptor as input, 14 machine learning (ML) methods were used to establish the model for pulmonary fibrosis prediction on the training set. These machine learning methods were provided by the AutoGluon framework (version 0.3.1; https://auto.gluon.ai/stable/index.html) and named as CatBoost, ExtraTreesEntr, ExtraTreesGini, KNeighborsDist, KNeighborsUnif, LightGBM, LightGBMLarge, LightGBMXT, NeuralNetFastAI, NeuralNetMXNet, RandomForestEntr, RandomForestGini, XGBoost, and Weighted Ensemble.

Fivefold cross validation was used as the training strategy to select the best model and the optimal hyper-parameters for each model. And the area under curve (AUC) was selected as the criterion for model evaluation. For the model using the given hyper-parameters, the average of AUC values during cross validation was calculated as its predictive power. The ML model that obtained the best results on the training cohort would be chosen to apply to the testing set. CT from patients diagnosed between May 2021 and March 2022 were used for model validation. All model implementations were done in the Python environment (version 3.7.3; https://www.python.org/).

### The diagnosis Performance of Radiologists

In the external validation set, each case was classified visually by three chest radiologists respectively with 20 years, 5 years, and 10 years of experience. Three radiologists independently evaluated the HRCT of patients in the external group without knowing the clinical information and diagnosis classification.

### Experimental Setting and Statistical Analysis

In our study, all HRCT images were considered for this binary classification task. Five random splits (80% training − 20% testing) were generated, ensuring that CT scans for the same patient were all grouped into either the training or the testing set, to do unbiased estimates of model evaluation. In each split, dimensionality reduction operations and model building with fivefold cross validation were executed on the training set, and the best model was selected via cross-validation results, validating on the testing set.

Since at least one CT scan was collected for each patient, model performance was validated both at the scan-level and patient-level. In this study, the result of the model at the patient-level was calculated by averaging the predicting results of all CT scans from the same patient. Continuous variables were expressed as means ± standard deviations. Identification ability of lung graph–based approach was assessed by using AUC, accuracy, sensitivity, specificity, the positive predictive value (PPV), and the negative predictive value (NPV). The performance lung graph–based ML model and chest radiologists in assessment of PPF was compared ROC. AUCs of external validation set were compared using DeLong’s test, and bootstrap (1000 times) was used to estimate 95% confidence intervals (CIs) of the above evaluation indicators.

## Results

### Population Characteristics

**Fig. 4:**
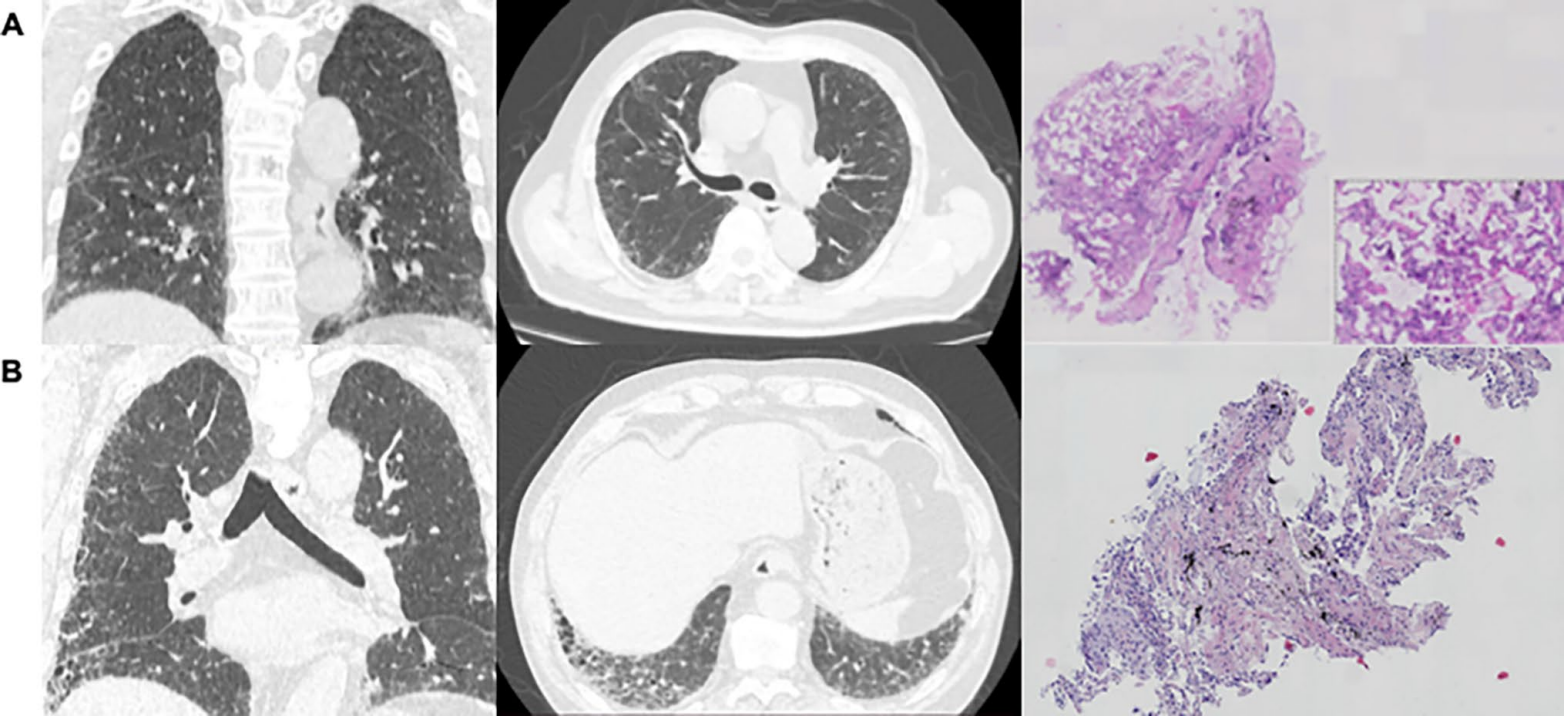
**A** A patient with a definite diagnosis of nonspecific interstitial pneumonia. CT showed non-fibrotic presentation of large bilateral pulmonary ground glass opacity with bilateral lower lung basal reticular pattern. Medium magnification of the lung showed lung tissue focal alveolar septum widening with lymphocyte and plasma cell infiltration, focal multinucleated giant cells, and alveolar epithelial hyperplasia, and no granulomas were seen. **B** A patient with a definite diagnosis of idiopathic pulmonary fibrosis. CT demonstrated fibrotic presentation of subpleural reticular pattern and ground glass opacity with honeycombing changes at the base of both lungs. Low magnification of the lung showed some of the alveolar septa widened with a little lymphocyte and plasma cell infiltration and alveolar epithelial hyperplasia; macrophages were seen in the alveolar lumen

During January 2018 to December 2021, 279 patients with ILD diagnosed by MDT at our hospital were included (156 f-ILD, 123 non-f-ILD). The median age of all included patients was 65 years (IQR, 59 to 71 years) and 160 (57.3%) were males. The median age was 67 years (IQR, 62 to 73 years) for f-ILD and 61 years (IQR, 55 to 68 years) for non-f-ILD. All included patients underwent one HRCT at least. A total of 417 HRCT images of 279 patients were included in the analysis. Figure 4 shows HRCT and corresponding pulmonary pathology of non-f-ILD and f- ILD. According to PFTs, there were 270 mild restrictive lung function (70–80% predicted), 111 moderate restrictive lung function (60–70% predicted), 28 moderately severe restrictive lung function (50–60% predicted), and 8 severe restrictive lung function (< 50% predicted).

### Identification of f-ILD on the Testing Set

After reducing the dimension, the final lung descriptor varied for each grouping split and contained an average number of 10 elements. Table 1 shows the predictive ability of the used 14 ML models on the training sets from five data splits. Among these models, the Weighted Ensemble obtained the best classification performance on the training set from each grouping split. Hence, the trained Weighted Ensemble models were applied to the corresponding testing sets from the five data splits for validating model robustness.

**Table 1:** Predictive ability of lung graph-based machine learning models to discriminate f-ILD on the training set

Lung graph-based ML Model	AUC					
	Split 1	Split 2	Split 3	Split 4	Split 5	Mean
Weighted Ensemble	**0.993**	**0.990**	**0.991**	**0.991**	**0.983**	**0.990**
CatBoost	0.989	0.985	0.988	0.989	0.977	0.986
ExtraTreesEntr	0.984	0.981	0.982	0.985	0.972	0.981
ExtraTreesGini	0.985	0.980	0.983	0.986	0.972	0.981
KNeighborsDist	0.955	0.922	0.921	0.916	0.914	0.926
KNeighborsUnif	0.959	0.923	0.923	0.920	0.910	0.927
LightGBM	0.989	0.986	0.983	0.984	0.973	0.983
LightGBMLarge	0.987	0.978	0.986	0.979	0.974	0.981
LightGBMXT	0.990	0.983	0.985	0.988	0.976	0.984
NeuralNetFastAI	0.991	0.990	0.990	0.990	0.982	0.989
NeuralNetMXNet	0.975	0.947	0.961	0.986	0.951	0.964
RandomForestEntr	0.981	0.978	0.985	0.984	0.971	0.980
RandomForestGini	0.982	0.976	0.987	0.986	0.972	0.981
XGBoost	0.986	0.982	0.989	0.983	0.975	0.983

The bold value indicates the best performance result on the training set. Split 1 to approximately 5 equals to five random splits of the data set (both training and testing set). AUC of the five random splits

*AUC* area under the curve, *ML* machine learning

**Fig. 5:**
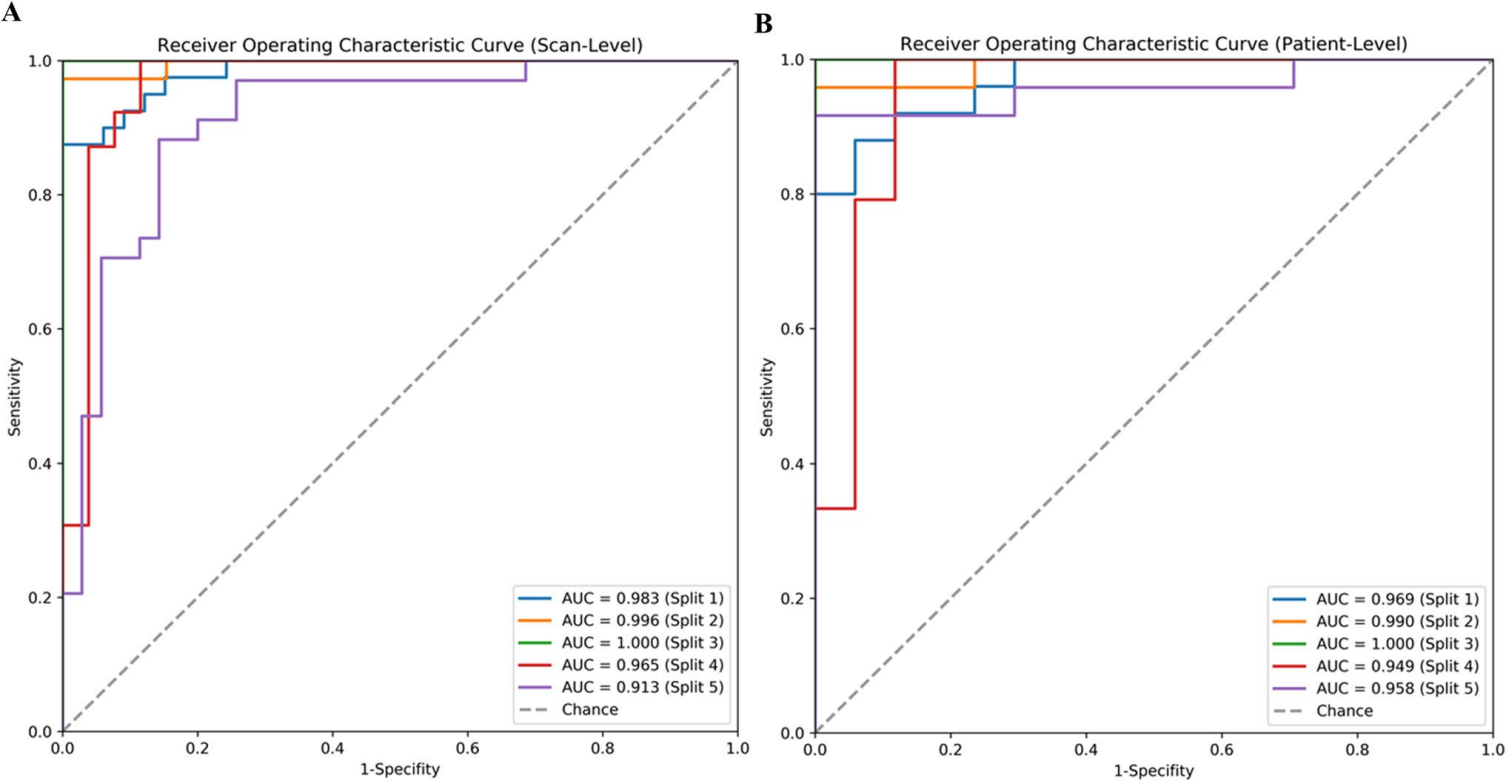
Receiver operating characteristic curves of the testing sets over five splits. **A** Scan level. **B** Patient level

To verify the robustness of the model, the trained Weighted Ensemble model was applied to the corresponding testing set from the five randomly divided data. Table 2 presents the mean and standard deviation of the performance evaluation metrics for the five randomly grouped models. At the CT sequence level, the lung graph–based machine learning model obtained good classification performance with an AUC value of 0.971 ± 0.032. The accuracy, sensitivity, and specificity of the model were 0.930 ± 0.057, 0.942 ± 0.040, and 0.921 ± 0.094, respectively. Similarly, the lung graph–based machine learning model showed good performance at the patient level with AUC values and accuracy of 0.973 ± 0.019 and 0.918 ± 0.059. The ROC curves for the five random groupings at the sequence level and patient level are shown in Fig. 5.

**Table 2:** Performance of the lung graph–based machine learning models in the identification of f-ILD on the testing set

Evaluation level	Method	AUC	Accuracy	Sensitivity	Specificity	PPV	NPV
Scan-level	Split 1	0.983	0.918	0.9	0.939	0.947	0.886
	Split 2	0.996	0.984	0.973	1	1	0.963
	Split 3	1	1	1	1	1	1
	Split 4	0.965	0.908	0.897	0.923	0.946	0.857
	Split 5	0.913	0.841	0.941	0.743	0.78	0.929
	Mean	0.971 ± 0.032	0.930 ± 0.057	0.942 ± 0.040	0.921 ± 0.094	0.935 ± 0.081	0.927 ± 0.051
Patient-level	Split 1	0.969	0.881	0.84	0.941	0.955	0.8
	Split 2	0.99	0.976	0.958	1	1	0.944
	Split 3	1	1	1	1	1	1
	Split 4	0.949	0.854	0.833	0.882	0.909	0.789
	Split 5	0.958	0.878	0.917	0.824	0.88	0.875
	Mean	0.973 ± 0.019	0.918 ± 0.059	0.910 ± 0.065	0.929 ± 0.068	0.949 ± 0.048	0.882 ± 0.081

All results are shown as mean values and standard deviations over the five random splits. Evaluation results (except AUC) of the proposed method were calculated by using the standard classification decision threshold of 0.5

*AUC* area under the curve, *PPV* positive predict value, *NPV* negative predict value

### External Validation of the Model and Comparison with Radiologists

Model performance was further evaluated in the independent validation set. At the CT sequence level, the diagnostic accuracy of the model radiologist A, radiologist B, and radiologist C was 0.968 (95% CI 0.926 to 1.000), 0.936 (95% CI 0.883 to 0.979), 0.830 (95% CI 0.755 to 0.904), and 0.894 (95% CI 0.830 to 0.947), respectively. The AUC values were 0.999 (95% CI 0.994 to 1.000), 0.933 (95% CI 0.879 to 0.979), 0.842 (95% CI 0.769 to 0.909), and 0.904 (95% CI 0.846 to 0.953), respectively. In addition, at the patient level, the diagnostic accuracy of the model radiologist A, radiologist B, and radiologist C was 0.986 (95% CI 0.959 to 1.000), 0.918 (95% CI 0.849 to 0.973), 0.822 (95% CI 0.726 to 0.904), and 0.904 (95% CI 0.836 to 0.973), respectively. The AUC values were 1.000 (95% CI 1.000 to 1.000), 0.917 (95% CI 0.855 to 0.973), 0.828 (95% CI 0.742 to 0.903), and 0.908 (95% CI 0.844 to 0.969), respectively. The sensitivity of the model was 0.971 (95% CI 0.912 to 1.000), and the specificity was 1.000 (95% CI 1.000 to 1.000) (Table 3).

**Table 3:** Performance of the lung graph–based machine learning model and radiologists in the identification of f-ILD on the independent validation set

Method	Evaluation level	AUC	Accuracy	Sensitivity	Specificity	PPV	NPV
Split 1	Scan-level	0.998 (0.992, 1.000)	0.957 (0.915, 0.989)	0.929 (0.837, 1.000)	0.981 (0.939, 1.000)	0.975 (0.917, 1.000)	0.944 (0.878, 1.000)
Split 2		0.997 (0.989, 1.000)	0.957 (0.915, 0.989)	0.905 (0.810, 0.979)	1.000 (1.000, 1.000)	1.000 (1.000, 1.000)	0.929 (0.855, 0.984)
Split 3		0.998 (0.992, 1.000)	0.968 (0.926, 1.000)	0.952 (0.880, 1.000)	0.981 (0.932, 1.000)	0.976 (0.914, 1.000)	0.962 (0.902, 1.000)
Split 4		0.997 (0.991, 1.000)	0.957 (0.915, 0.989)	0.905 (0.814, 0.978)	1.000 (1.000, 1.000)	1.000 (1.000, 1.000)	0.929 (0.862, 0.983)
Split 5		0.995 (0.984, 1.000)	0.968 (0.926, 1.000)	0.929 (0.844, 1.000)	1.000 (1.000, 1.000)	1.000 (1.000, 1.000)	0.945 (0.879, 1.000)
Average		0.999 (0.994, 1.000)	0.968 (0.926, 1.000)	0.929 (0.844, 1.000)	1.000 (1.000, 1.000)	1.000 (1.000, 1.000)	0.945 (0.873, 1.000)
Radiologist A		0.933 (0.879, 0.979)	0.936 (0.883, 0.979)	0.905 (0.810, 0.970)	0.962 (0.902, 1.000)	0.950 (0.868, 1.000)	0.926 (0.849, 0.983)
Radiologist B		0.842 (0.769, 0.909)	0.830 (0.755, 0.904)	0.952 (0.882, 1.000)	0.731 (0.607, 0.854)	0.741 (0.621, 0.857)	0.950 (0.871, 1.000)
Radiologist C		0.904 (0.846, 0.953)	0.894 (0.830, 0.947)	1.000 (1.000, 1.000)	0.808 (0.692, 0.906)	0.808 (0.690, 0.906)	1.000 (1.000, 1.000)
Split 1	Patient-level	1.000 (1.000, 1.000)	0.986 (0.959, 1.000)	0.971 (0.905, 1.000)	1.000 (1.000, 1.000)	1.000 (1.000, 1.000)	0.974 (0.915, 1.000)
Split 2		0.997 (0.988, 1.000)	0.973 (0.932, 1.000)	0.943 (0.861, 1.000)	1.000 (1.000, 1.000)	1.000 (1.000, 1.000)	0.950 (0.881, 1.000)
Split 3		0.999 (0.995, 1.000)	0.986 (0.959, 1.000)	0.971 (0.903, 1.000)	1.000 (1.000, 1.000)	1.000 (1.000, 1.000)	0.974 (0.913, 1.000)
Split 4		0.998 (0.994, 1.000)	0.959 (0.918, 1.000)	0.914 (0.821, 1.000)	1.000 (1.000, 1.000)	1.000 (1.000, 1.000)	0.927 (0.838, 1.000)
Split 5		0.998 (0.992, 1.000)	0.973 (0.932, 1.000)	0.943 (0.857, 1.000)	1.000 (1.000, 1.000)	1.000 (1.000, 1.000)	0.950 (0.870, 1.000)
Average		1.000 (1.000, 1.000)	0.986 (0.959, 1.000)	0.971 (0.912, 1.000)	1.000 (1.000, 1.000)	1.000 (1.000, 1.000)	0.974 (0.919, 1.000)
Radiologist A		0.917 (0.855, 0.973)	0.918 (0.849, 0.973)	0.886 (0.774, 0.974)	0.947 (0.872, 1.000)	0.939 (0.853, 1.000)	0.900 (0.795, 0.977)
Radiologist B		0.828 (0.742, 0.903)	0.822 (0.726, 0.904)	0.971 (0.912, 1.000)	0.684 (0.525, 0.825)	0.739 (0.608, 0.860)	0.963 (0.880, 1.000)
Radiologist C		0.908 (0.844, 0.969)	0.904 (0.836, 0.973)	1.000 (1.000, 1.000)	0.816 (0.688, 0.938)	0.833 (0.705, 0.944)	1.000 (1.000, 1.000)

Statistics in the square brackets showed 95% confidence intervals (CIs). Evaluation results (except AUC) of the proposed method were calculated by using the standard classification decision threshold of 0.5

*Average* average of five groups of models, *PPV* positive predict value, *NPV* negative predict value

**Fig. 6:**
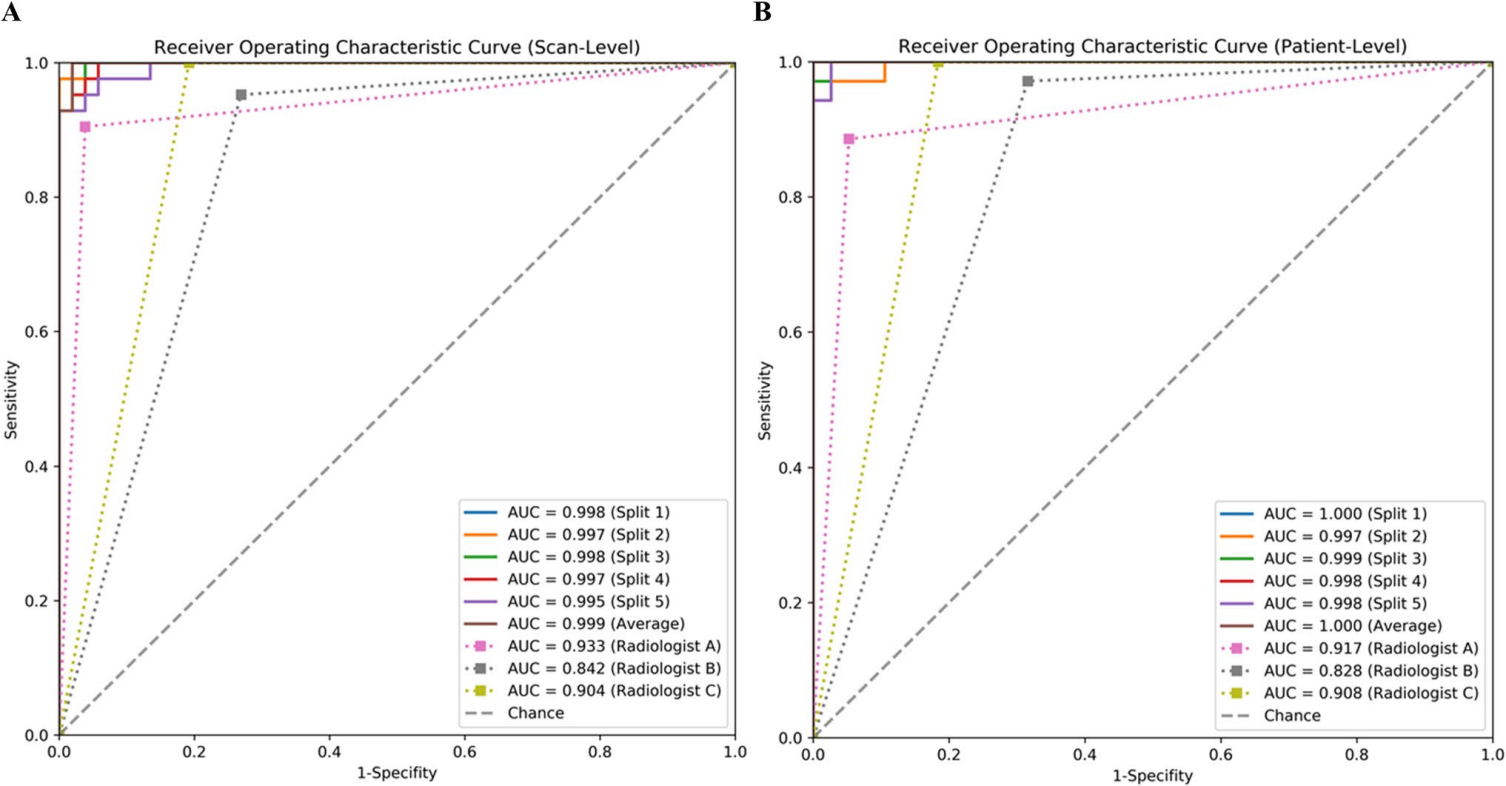
Receiver operating characteristic curves of the external validation set. **A** Scan level. **B** Patient level. Average, average of five groups of models

The diagnostic performance of the model was superior to that of the radiologist, both at the CT sequence level and at the patient level, and there was a statistically significant difference in AUC values between the model and radiologist A, radiologist B, and radiologist C (*p* < 0.05) (Table 4). The corresponding ROC curves are shown in Fig. 6.

**Table 4:** AUC comparisons between the lung graph–based model and visual assessments provided by three radiologists

Evaluation level	*p*-values			
	Method	Radiologist A	Radiologist B	Radiologist C
Scan-level	Split 1	0.0160	< 0.0001	0.0006
	Split 2	0.0183	< 0.0001	0.0007
	Split 3	0.0160	< 0.0001	0.0007
	Split 4	0.0153	< 0.0001	0.0008
	Split 5	0.0242	< 0.0001	0.0011
	Average	0.0142	< 0.0001	0.0006
Patient-level	Split 1	0.0111	< 0.0001	0.0038
	Split 2	0.0156	< 0.0001	0.0049
	Split 3	0.0121	< 0.0001	0.0042
	Split 4	0.0122	< 0.0001	0.0047
	Split 5	0.0131	< 0.0001	0.0047
	Average	0.0111	< 0.0001	0.0038

All values were *p*-values obtained by Delong’s test. These values < 0.05 were considered significant

*Average* average of five groups of models

## Discussion

To our knowledge, this is the first study to develop and validate a lung graph–based machine learning model for identification of f-ILD, and the lung graph-based Weighted Ensemble model exhibited excellent classification performance in the validation set.

ILD is characterized by varying degrees of inflammation and fibrosis in interstitial lung [1]. Compared to patients with non-f-ILD, patients with f-ILD had a poorer quality of life and prognosis. Imaging characteristics of f-ILD tend to be dominated by reticular pattern and traction bronchiectasis with or without honeycombing [3]. Whereas non-f-ILD mainly presents as ground glass opacity or consolidation. However, definite diagnosis depends on tissue pathology. The treatment and prognosis of f-ILD and non-f-ILD are significantly different; early and accurate identification is especially essential to improve the prognosis [26–28]

Rafaee et al. [29] achieved for the first time the identification of UIP in patients with IPF based on handcrafted radiomics with an AUC of 0.66. However, this artificial labeling of regions of interest was time- and effort-consuming, and the diagnostic performance of handcrafted radiomics-based model and physician remains unknown. The underlying structure of the lung map model used in this study is based on the 3D morphology of the lungs, dividing the lung into different regions, extracting each node and coding for different nodes, filtering features according to their importance, and analyzing the correlation between different features. Unlike previous studies that selected only a few levels of images and based on regions of interest [30–32], the 3D lung graph combined with deep learning algorithms achieved the integration from local analysis to 3D images. The potential of graph model–based disease classification was confirmed in various disease such as chronic thromboembolic pulmonary hypertension, multidrug resistance prediction, and pulmonary tuberculosis type [23, 24, 33]. In our research, classification method divided the lung region into different sub-regions and completed the overall analysis to encode the subtle differences between different types of ILD by considering the feature nodes of the associated sub-regions and analyzing the global radiomics feature distribution. The performance of the model at both patient level and CT sequence level was considered. Our lung graph–based method gained good diagnostic accuracy in patient level and CT scan level in the testing set. Further, the external validation cohort demonstrated the excellent performance of the model, achieving higher diagnostic accuracy compared to radiologists.

There are several limitations in this study. First, more studies are needed to verify this result for a better clinical interpretation, depending on more diverse data. Second, although this classification model achieved f-ILD screening, it could not realize the segmentation and quantitative analysis of specific lesions. Third, the performance of the deep learning model was compared to only three chest radiologists in different experiences, which may not fully represent the entire range of physician capabilities, but as a national respiratory medicine center, the radiologists are likely to be more experienced compared to most hospitals and may overestimate the diagnostic capabilities of the physicians. Finally, the model is helpful to dichotomize only f-ILD and non-f-ILD without performing disease classification for other different types of ILD, and this is the next major step of our further study. Moreover, the correlation of quantitative fibrosis and restrictive lung dysfunction need further research.

## Conclusions

The lung graph-based machine learning model achieved high accuracy in identifying f-ILD. This model improves the efficiency of f-ILD diagnosis which could aid clinicians to accurately assess ILD.

## Data Availability

The original contributions presented in the study are included in the article/Supplementary Materials; further inquiries can be directed to the corresponding author.

## Abbreviations

ILD: Interstitial lung disease
f-ILD: Fibrotic interstitial lung disease
Non-f-ILD: Non fibrotic interstitial lung disease
IPF: Idiopathic pulmonary fibrosis
NSIP: Nonspecific interstitial pneumonia
CTD-ILD: Connective tissue disease-associated interstitial lung disease
COP: Cryptogenic organizing pneumonia
HRCT: High resolution computed tomography
ML: Machine learning
AUC: Area under the curve
PPV: Positive predict value
NPV: Negative predict value
